# Differential activation of monocytes and PMNs in orofacial granulomatosis patients induced by bacterial and non-bacterial stimuli

**DOI:** 10.3389/fimmu.2025.1522495

**Published:** 2025-05-06

**Authors:** Francesco Palestra, Luca Modestino, Annagioia Ventrici, Arianna Monteforte, Gina Memoli, Anne Lise Ferrara, Leonardo Cristinziano, Remo Poto, Francesca Wanda Rossi, Gilda Varricchi, Amato De Paulis, Gianni Marone, Giuseppe Spadaro, Angelica Petraroli, Stefania Loffredo, Maria Rosaria Galdiero

**Affiliations:** ^1^ Department of Translational Medical Sciences, University of Naples Federico II, Naples, Italy; ^2^ Department of Internal Medicine and Clinical Immunology, University Hospital of Naples Federico II, Naples, Italy; ^3^ Center for Basic and Clinical Immunology Research (CISI), WAO Center of Excellence, University of Naples Federico II, Naples, Italy; ^4^ Institute of Experimental Endocrinology and Oncology, National Research Council (CNR), Naples, Italy

**Keywords:** cytokines/chemokines, ROS, NETs, bacterial stimuli, inflammation, immunopathogenesis

## Abstract

**Introduction:**

Orofacial Granulomatosis (OFG) is a rare chronic inflammatory disorder characterized by persistent or recurrent swelling of the lips and oral mucosa, often accompanied by granulomatous inflammation in the orofacial region with limited effective treatment options available. Emerging evidence suggests an immune dysregulation in the development and progression of OFG. Immune cells, including monocytes and neutrophils (PMNs), are involved in autoimmune and inflammatory diseases by releasing pro-inflammatory and immunomodulatory molecules.

**Methods:**

Considering that microbial agents have been suggested as potential triggers for OFG, in this study we evaluated the effect of LPS, fMLP and PMA on the activation of monocytes and PMNs purified by 11 OFG patients and 11 sex-and age-matched healthy donors (HDs).

**Results:**

Upon stimulation, OFG-derived monocytes displayed a higher release of pro-inflammatory cytokines (CXCL8/IL-8, IL-6, TNF-α, IL-33) compared to HDs. Conversely, OFG-derived monocytes showed a lower release of IL-10, IFN-γ compared to HDs. Upon stimulation, peripheral PMNs from OFG patients released large amounts of TNF-α and MPO compared to HDs. In addition, OFG-derived PMNs showed high percentages of activated PMNs (CD62L^-^) and increased ROS production compared to HDs. Compared to HDs, OFG patients presented higher serum levels of MMP-9, MPO and TNF-α, together with MPO-DNA complexes and citrullinated histone H3 (CitH3) (two biomarkers for neutrophil extracellular traps).

**Discussion:**

These preliminary data suggest that in presence of various stimuli, monocytes and PMNs of OFG patients displayed an activated phenotype compared to HDs. Unraveling the interplay between bacterial triggers and immune cell function in OFG will be necessary to elucidate mechanisms driving this complex disease and identify novel therapeutic targets for improved management of OFG patients.

## Introduction

Orofacial granulomatosis (OFG) is a rare chronic inflammatory disorder characterized by persistent and/or recurrent granulomatous inflammation affecting the soft tissues of the oral and maxillofacial regions (1). OFG is often associated with other granulomatous diseases, such as Crohn’s disease, highlighting its complex pathogenesis and potential systemic implications (1, 2). Despite its clinical significance, OFG remains a poorly understood condition, lacking standardized diagnostic criteria and treatment guidelines (1).

The pathogenesis of OFG remains elusive, with a complex interplay of genetic, environmental, and immunological factors thought to contribute to its development (1, 3). Evidence suggests a potential role for immune cells in driving granulomatous inflammation in OFG (4, 5). In fact, granulomas are aggregates of immune cells composed of macrophages, lymphocytes, and multinucleated giant cells playing a central role in the inflammatory response in OFG (1, 4, 5). More in detail, activated macrophages release pro-inflammatory cytokines and chemokines that recruit other immune cells to the site of inflammation contributing to the formation and maintenance of granulomas in OFG (6).

Monocytes and neutrophils (PMNs), components of the innate immunity, play crucial roles in the initiation, regulation, and resolution of inflammation (7–9). Monocytes exhibit distinct subsets (classical, intermediate, and non-classical) and functions in inflammation and tissue repair (10). Monocytes act in immune regulation processes releasing both pro-inflammatory (CXCL8/IL-8, IL-6, TNF-α) and anti-inflammatory (IL-10) cytokines, depending on the physio-pathological context (7, 11).

PMNs are the first cells that react in case of infection (12, 13), attracted by chemotactic stimuli [e.g., formylated peptides, CXCL8/IL-8, lipopolysaccharide (LPS)] released during the inflammatory process (14). At the inflammatory site, PMNs strategies to kill the microorganisms include degranulation, phagocytosis, the production of reactive oxygen species (ROS) (15) and Neutrophil Extracellular Traps (NETs), a mesh-like structures composed by nuclear components (DNA and histones) and granular proteins (14, 16, 17).

The aim of this study was to investigate the potential role of monocytes and PMNs in OFG pathogenesis. Considering that microbial agents have been suggested as potential triggers for OFG (4, 18, 19), we evaluated the effect of bacterial stimuli like LPS and *N*-Formylmethionyl-leucyl-phenylalanine (fMLP) as well as the effect of a non-immunological stimulus, such as the phorbol 12-myristate 13-acetate (PMA) on the activation of monocytes and PMNs of healthy donors (HDs) and OFG patients.

## Materials and methods

### Patient recruitment

In this study, 11 OFG patients, were prospectively recruited at the Division of Clinical Immunology and Internal Medicine of the University of Naples Federico II (Naples, Italy). Patients were eligible for enrollment in the study if they were aged 18–70 years and had a diagnosis of OFG based on clinical and histopathological features. The main exclusion criteria were other autoimmune diseases, infections, and malignancies. Peripheral blood samples were collected at the Center for Basic and Clinical Immunology Research (CISI), University of Naples Federico II and were immediately processed. Serum samples were obtained (+4°C, 400 ×g, 20 min) and stored (−80°C) until used. Moreover blood samples of 11 healthy controls (Healthy Donors – HDs), matched for sex and age, were collected at Center for Basic and Clinical Immunology Research (CISI), University of Naples Federico II. The study was approved by the Ethics Committee of the University of Naples Federico II (n. 319/17) and conducted in accordance with Good Clinical Practice (GCP) guidelines and adhered to the Declaration of Helsinki. Written informed consent was obtained from all participants.

### Reagents

The following reagents were used for the experiments: antibiotic solution (10.000 IU/ml penicillin, 10 mg/mL streptomycin) (Lonza, Basel, Switzerland), RPMI 1640 (Microgem, Naples, Italy). L-glutamine, fetal bovine serum (FBS), Dextran, Ficoll-Paque Histopaque-1077 Percoll density gradient, LPS (from Escherichia coli serotype 026:B6), fMLP and PMA (Sigma Aldrich, St. Louis, MO, USA). EasySep Neutrophil Enrichment Kit (StemCell Technologies, Vancouver, Canada).

### Isolation of monocytes and neutrophils

Cells were isolated from whole blood of HDs and OFG patients (HBsAg−, HCV−, and HIV−). Leukocytes were separated from erythrocytes by dextran sedimentation. PBMCs and PMNs were purified by Ficoll-Paque Histopaque-1077 density gradient centrifugation (400 ×g for, 20 min at 22 ˚C). Then, monocytes were purified from PBMCs with CD14 microbeads (Miltenyi Biotec, Bergisch Gladbach, Germany), according to the manufacturer’s protocol (20). PMNs obtained after Ficoll-Paque were washed and purified by Percoll (65%) density gradient centrifugation (660 ×g for 20 minutes at 22°C), as previously described (21). Finally, neutrophils were isolated from granulocytes (to reach >99% purity) by negative selection using the EasySep Neutrophil Enrichment Kit (22). These cells were >99% neutrophils as evaluated by flow cytometric analysis with the following antibodies: anti-CD3, anti-CD14, anti-CD15, anti-CD11b, anti-CD193 (Miltenyi Biotec), anti-CD62L (L-selectin) (BD Biosciences, San Jose, CA, USA) and anti-CD66b (BioLegend, San Diego, CA, USA). Samples were analyzed on the MACSQuant Analyzer 10 (Miltenyi Biotec) and in the FlowJo software, v.10. Dead cells, doublets, debris and eosinophils were excluded from the analysis. Data were expressed as percentage of positive cells or median fluorescence intensity (23).

### Cell cultures

Monocytes (10^6^ cells per well) of HDs and OFG patients were seeded in 24-well plate and stimulated with LPS (100 ng/mL), fMLP (1 µM), PMA (10 ng/mL) or medium alone for 18h at 37°C with 5% CO_2_. At the end of incubation, supernatants were harvested, centrifuged (500 ×g, 4°C, 10 min), and stored at −80°C until use. Lysis of the remaining cells in the plates was carried out using 0.1% Triton X-100 for total protein quantification by a Bradford assay (Bio-Rad Laboratories, Segrate MI, Italy). PMNs (1.5 × 10^5^ cells) were seeded in 96-well plate and stimulated with LPS (100 ng/mL), fMLP (1 µM) or PMA (10 ng/mL), for 1h at 37°C with 5% CO_2_. At the end of incubation, supernatants were collected and stored at -80°C until use.

### ELISA assays

The release of soluble mediators in the supernatants was measured in duplicate using commercially available ELISA kits for CXCL8/IL-8, IL-6, TNF-α, IL-33, IL-10, IFN-γ, MMP-9 and MPO (R&D Systems, Minneapolis, MN, USA). Since the number of monocytes can vary among wells and different experiments, the results from monocytes were normalized for the total protein content in each well, determined in the cell lysates and expressed as ng/mg of total proteins. The absorbance of the sample was measured at 450 nm using a microplate reader (Tecan, Grödig, Austria). The ELISA detection ranges are available on R&D website.

### Flow cytometry

PMNs were kept in RPMI with 10% FBS and antibiotics for 30 min at 37°C, then were washed with PBS and stimulated with LPS (100 ng/mL), fMLP (1 µM), PMA (10 ng/mL) and/or RPMI 1640 medium with 5% of FBS (Euroclone, Milan, Italy), as negative control, for 1h at 37°C. 1.5 × 10^5^ PMNs of OFG patients and HDs, were seeded in U-shaped 96-well plate and Zombie Violet dye (BioLegend, San Diego, CA, USA) was added to evaluate cell viability (20 min, +4°C). Then, PMNs were stained (20 min, +4°C) in PBS containing 1% FBS with the following antibodies: APC-conjugated anti-CD66b (clone REA306, dilution 1:50), VioBlue-conjugated anti-CD193 (clone REA574, dilution 1:10), PerCP-conjugated anti-CD11b (clone REA713, dilution 1:50) and FITC-conjugated anti-CD62L (clone 145/15, dilution 1:10) from Miltenyi Biotech. Cells were acquired by MACS Quant Analyzer 10 (Miltenyi Biotec) and analyzed by FlowJo v.10 software. Doublets and debris (identified based on forward and side scatter properties), dead cells (identified with Zombie Violet Fixable Viability Kit; BioLegend) and eosinophils (identified based on the CCR3+ exclusion gate) were excluded from the analysis. The complete flow cytometry gating strategy used to identify PMNs from peripheral blood samples of HDs and OFG patients is shown in the **Supplementary Figure 1**.

### Reactive oxygen species production

PMNs (2 × 10^6^ cells/mL) were resuspended in the RPMI 1640 medium with 10% of FBS and antibiotics and incubated for 30 min at 37°C. Then, PMNs were washed with PBS solution and were incubated for 30 min with 2’,7’-dichlorodihydrofluorescein diacetate (H_2_DCF-DA) (10µg/mL) (Life Technologies, Carlsbad, CA, USA). At the end of incubation, PMNs were washed in PBS and resuspended in RPMI 5% FBS in the presence or absence of LPS (100 ng/mL), fMLP (1 µM) or PMA (10 ng/mL) and analyzed with EnSpire Multimode Plate Reader (Perkin Elmer, Waltham, MA, USA). DCF mean fluorescence intensity was measured at an excitation wavelength of 492–495 nm and emission at 517–527 nm. The ability of LPS, fMLP and PMA to induce ROS formation was measured as compared to negative control.

### Serum NET biomarkers detection

Serum MPO-DNA complexes levels of OFG patients and HDs, were measured as previously described (24). Briefly, MaxiSorp 96-well microplates were coated with the mouse monoclonal anti-human MPO antibody (5 mg/mL; Bio-Rad) diluted in PBS and incubated overnight at +4°C then blocked with 1% of BSA in PBS. The samples and the anti-DNA-POD antibody of the Cell Death Detection ELISA kit (Roche, Basel, Swiss) were added to the wells for 2 hours at 22°C, respectively. After incubations, substrate solution from Cell Death Detection ELISA kit was added, and the absorbance was measured at 405 nm with a microplate reader (Tecan, Grödig, Austria). The concentration of Citrullinated Histone H3 (CitH3), a specific NET biomarker (25), was measured in sera of OFG patients and HDs, using the ELISA kit developed by Cayman Chemicals (Ann Arbor, MI, USA) according to the manufacturer’s instructions. The absorbance of CitH3 was determined at 450 nm. The ELISA sensitivity range was 0.15 – 10 ng/mL.

### Statistical analysis

Statistical analysis was performed using GraphPad Prism 9 (GraphPad Software, San Diego, CA, USA). The data are expressed as mean values ± standard deviation (SD) of the indicated number of experiments. Data normality was assessed using the D’Agostino & Pearson normality test. If data were normally distributed at a 0.05 significance level, parametric tests were applied. For non-normally distributed data, nonparametric tests were used. Group comparisons were made using Student’s t-test or Mann–Whitney U test, depending on the parametric or nonparametric distribution of the continuous variables. Repeated measures one-way or two-way ANOVA were used where appropriate, as described in the figure legends. A p-value ≤ 0.05 was considered statistically significant.

## Results

### Patients

In this study, 11 OFG patients and 11 HDs, matched for sex and age, were recruited at the Division of Allergy and Clinical Immunology of the University of Naples Federico II (Naples, Italy). Of 11 patients, 4 were males (36.4%) and 7 were females (63,6%). Regarding the HDs, 3 were males (27.3%) and 8 were females (72.7%). Eligible patients were aged 18–70 years and had a diagnosis of OFG based on clinical and histopathological criteria. The mean age at recruitment was 36.6 (± 17.1) for patients and 38.5 (± 16.7) for HDs. The mean age at diagnosis was 32 (± 18.3) years, whereas the mean age of onset was 34 (± 17) with a mean diagnostic delay of 3.4 years (± 3.6). None of the patients presented familial history for OFG or its variants. Among all the patients, all (100%) displayed persistent swelling of one or both lips, which was the most frequent localization. Additionally, 2 patients (18.2%) also presented oral aphthosis and 2 patients (18.2%) cervical lymphadenopathy. Histological evaluation was available for 7/11 patients (63.6%), and in 3/7 (42.8%), non-caseating granulomas were found. In the remaining 4 patients (57.1%), chronic non-specific inflammation was described, without pathognomonic diagnostic elements. Two patients (18.2%) also presented neurological symptoms (hemicranias, dizziness). Five (45.5%) patients complained gastrointestinal symptoms (diarrhea, abdominal pain), even in absence of Inflammatory Bowel Disease (IBD). Food or inhalant allergic sensitization was found in 5 patients (45.5%), by skin prick test (SPT) or specific IgE for food or inhalant allergens. In all these 5 patients, a history of clinical manifestations of allergic rhinitis, asthma or food allergy was described.

Among the enrolled patients, only one patient was treatment-naïve, 36.3% were under local steroids, 45.4% were receiving low-dose oral corticosteroids (OCS), none of these patients was on DMARD therapy. One patient was under bDMARDs (adalimumab). With regards of co-morbidities, among OFG patients, 3 suffered from systemic arterial hypertension (27.3%), and 1 patient suffered from metabolic syndrome (9,1%). Among OFG patients with hypertension, 2 out of 3 were under ACE-inhibitors and one patient was under the combination ACE inhibitor/thiazide diuretic. Among the HDs, none suffered from atopy, or metabolic syndrome. 3 HD suffered from obesity (27,3%) and 3 suffered from systemic arterial hypertension (27,3%). Of these 3 patients, all were under the combination ACE inhibitors/thiazide diuretics. The clinical characteristics of the study population are shown in **Table 1**.

**Table 1:** Demographic and clinical features of OFG patients and healthy donors.

Characteristics	OFG Patients	Healthy Donors
Number, n (%)	11 (100)	11 (100)
Sex, n (%)		
- Males	4 (36,4)	3 (27,3)
- Females	7 (63,7)	8 (72,7)
Age at recruitment, years		
Mean ± SD	36,6 ± 17,1	38,5 ± 16,7
Age at diagnosis, years		
Mean ± SD	32 ± 18,3	NA
Age at onset, years		
Mean ± SD	34 ± 16,5	NA
Signs and symptoms, n (%)		
Lip swelling (one/both)	11 (100)	0 (0)
Neurological symptoms	2 (18,2)	0 (0)
Atopy	5 (45,5)	0 (0)
Gastrointestinal symptoms	5 (45,5)	0 (0)
Oral aphthosis	2 (18,2)	0 (0)
Cervical lymphoadenopathy	2 (18,2)	0 (0)
Allergy work up, n (%)		
Skin Prick Test	5 (45,4)	NA
Positive	5 (100)	NA
Therapeutic options, n (%)		
Oral steroids	5 (45,4)	NA
Intralesional Steroids	4 (36,3)	NA
Anti-H1 Drugs	4 (36,3)	NA
Biological Drugs	1 (9,1)	NA
Comorbidities		
- Obesity	0	3 (27,3)
- Hypertension	3 (27,3)	3 (27,3)
- Metabolic Syndrome	1 (9,1)	0

NA, Not Available.

### Effect of LPS, fMLP and PMA on activation of monocytes of OFG patients

**Figure 1:**
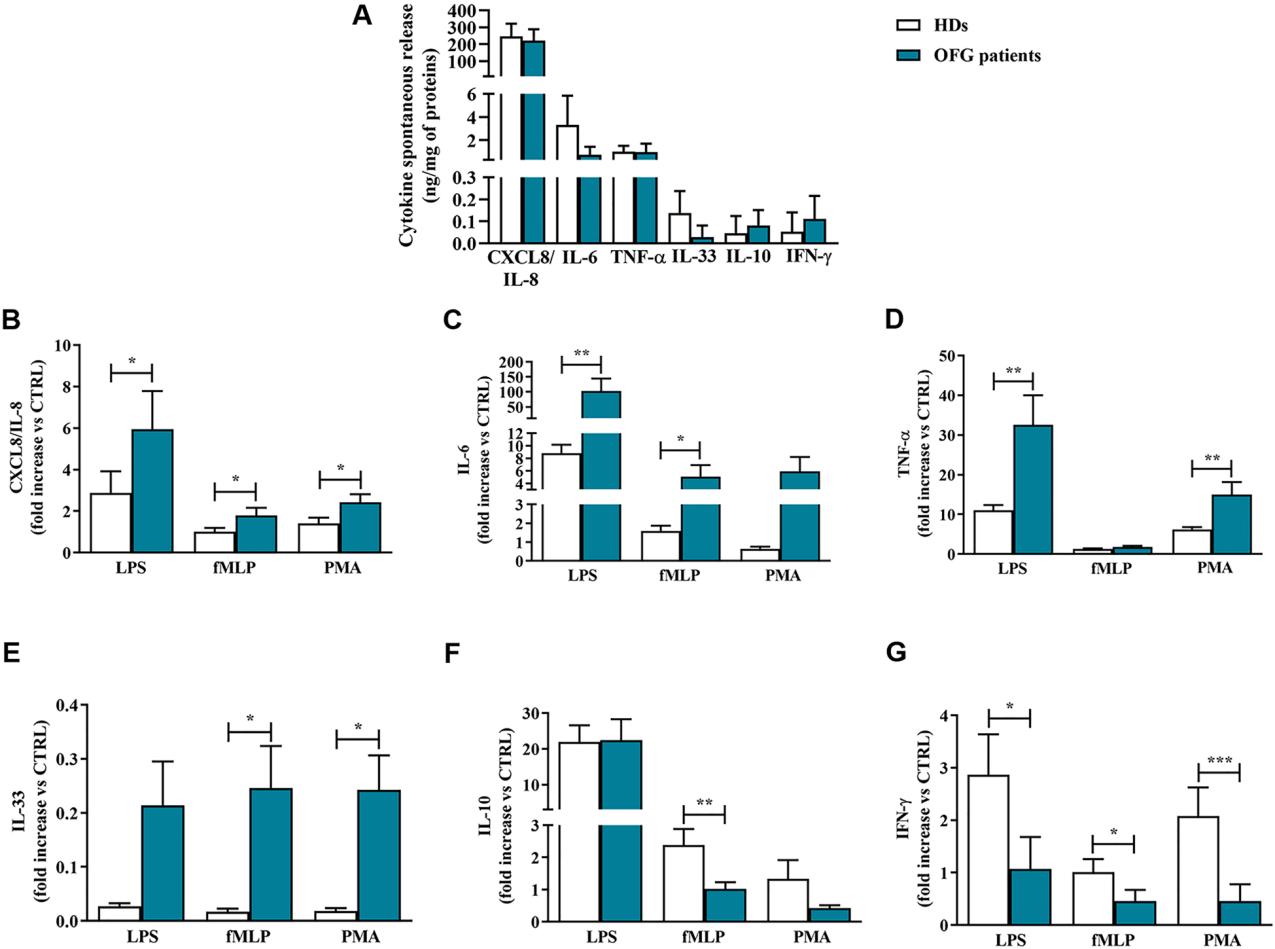
**(A)** Monocytes (1 x 10^6^ cells/well) were incubated for 18 hrs at +37°C with 5% CO_2_ in complete medium. At the end of incubation, the concentrations of CXCL8/IL-8, IL-6, TNF-α, IL-33, IL-10, and IFN-γ proteins in supernatants were evaluated by ELISA and expressed as ng of mediators for mg of total proteins. **(B-G)** Monocytes (1 x 10^6^ cells/well) were incubated for 18 hrs at +37°C with 5% CO_2_ with complete medium, LPS (100 ng/mL), fMLP (1 µM) or PMA (10 ng/mL). CXCL8/IL-8 **(B)**, IL-6 **(C)**, TNF-α **(D)**, IL-33 **(E)**, IL-10 **(F)** and IFN-γ **(G)** proteins in supernatants were evaluated by ELISA and normalized for the total protein content in each well. Effect of LPS, fMLP, and PMA on the mediators release were expressed as fold increase *vs* control. Data are expressed as mean ± SD. All the experiments were run in duplicate. *p < 0.05; **p < 0.01; ***p < 0.005 *vs*. the same condition of respective HDs.

Upon activation, monocytes release large amount of several mediators (26–28). However, after 18 hrs of incubation they spontaneously secreted a certain amount of these molecules including CXCL8/IL-8, IL-6, TNF-α, IL-33, IL-10, and IFN-γ (**Figure 1A**). There were no differences between the spontaneous release of the cytokines/chemokines in monocytes purified by HDs and OFG patients (**Figure 1A**). Next, we evaluated the effects of LPS (100 ng/mL), fMLP (1 µM) or PMA (10 ng/mL) on the secretion of pro-inflammatory and anti-inflammatory mediators from monocytes of OFG patients compared with that of HDs. **Figure 1** shows that LPS, fMLP, and PMA induced cytokine/chemokine release from monocytes of both HDs and OFG patients. More in details, upon activation by all tested stimuli (LPS, fMLP and PMA), OFG-derived monocytes displayed a higher release of CXCL8/IL-8 (**Figure 1B**) and IL-6 (**Figure 1C**) compared with monocytes from HDs. TNF-α (**Figure 1D**) release induced by LPS and PMA, and IL-33 (**Figure 1E**) release induced by fMLP and PMA, were higher in monocytes of OFG patients than that of HDs. Conversely, monocytes from OFG patients presented a lower release of the anti-inflammatory cytokine IL-10 (**Figure 1F**) under fMLP and IFN-γ (**Figure 1G**) under all tested stimuli compared with monocytes purified from HDs.

### Degranulation and cytokines release of PMNs in OFG patients

**Figure 2:**
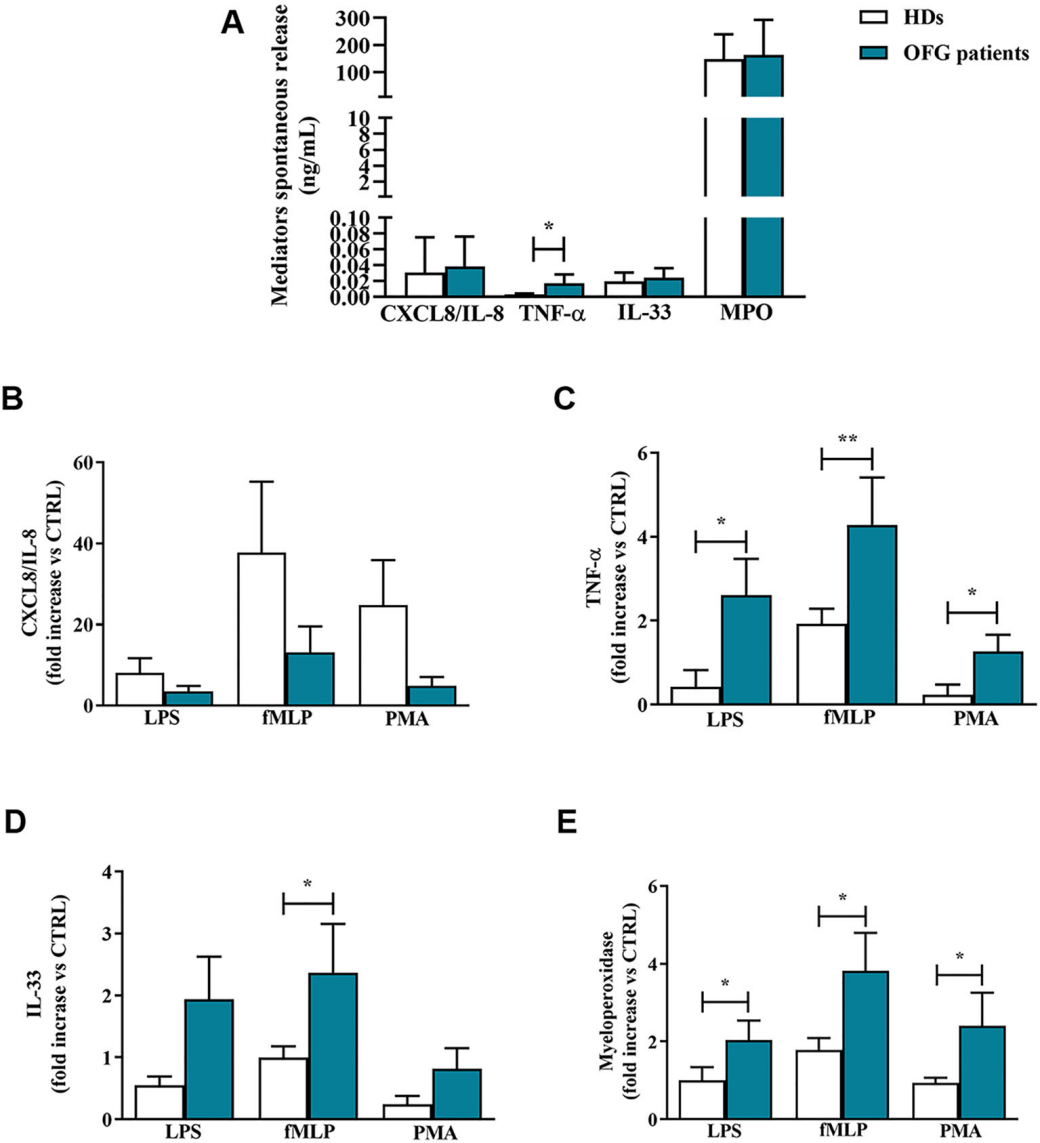
**(A)** PMNs (1.5 x 10^5^ cells/well) were incubated for 1 h at +37°C with 5% CO_2_, in complete medium. At the end of incubation the supernatants were harvested and the extracellular levels of CXCL8/IL-8, TNF-α, IL-33 and MPO were measured by ELISA. **(B–E)** PMNs (1,5 x 10^5^ cells/well) were incubated with complete medium, LPS (100 ng/mL), fMLP (1 µM) or PMA (10 ng/mL) for 1 h at +37°C with 5% CO_2_. At the end of incubation the supernatants were harvested and the extracellular levels of CXCL8/IL-8 **(B)**, TNF-α **(C)**, IL-33 **(D)** and MPO **(E)** were evaluated by ELISA. Effect of LPS, fMLP, and PMA on the mediator release was expressed as fold increase *vs* control. Results were expressed as mean ± SD. All the experiments were run in duplicate. *p < 0.05; **p < 0.01 *vs*. the same condition of respective HDs.

It has been shown that neutrophils, upon stimulation, were capable to release a large amount of granular enzymes and cytokines (29). Here, we evaluated the spontaneous and the induced release, upon stimulation with LPS, fMLP and PMA, of some neutrophil-related mediators in OFG patients and HDs PMNs. Within the control medium, after 1 h of incubation, PMNs purified from peripheral blood of OFG patients and HDs, released a certain amount of cytokines including TNF-α, IL-33, CXCL8/IL-8 (**Figure 2A**). PMNs of OFG patients presented a higher spontaneous release of TNF-α compared to their counterpart of HDs. No differences were found in the spontaneous release of IL-33, CXCL8/IL-8 and MPO between OFG patients and HDs (**Figure 2A**). After 1h of stimulation, CXCL8/IL-8 release induced by LPS, fMLP, and PMA was slightly more pronounced in PMNs from HDs than that of OFG patients (**Figure 2B**). By contrast, under stimulation with LPS, fMLP and PMA, PMNs from OFG patients released a higher quantity of TNF-α (**Figure 2C**) compared to PMNs from HDs. IL-33 release was higher in PMNs of OFG patients only under fMLP stimulation (**Figure 2D**). Finally, under the activation by all the tested stimuli, higher levels of myeloperoxidase (MPO) were released by OFG PMNs compared with HD PMNs (**Figure 2E**).

### Serum levels of neutrophil-related mediators and NET biomarkers in OFG patients

**Figure 3:**
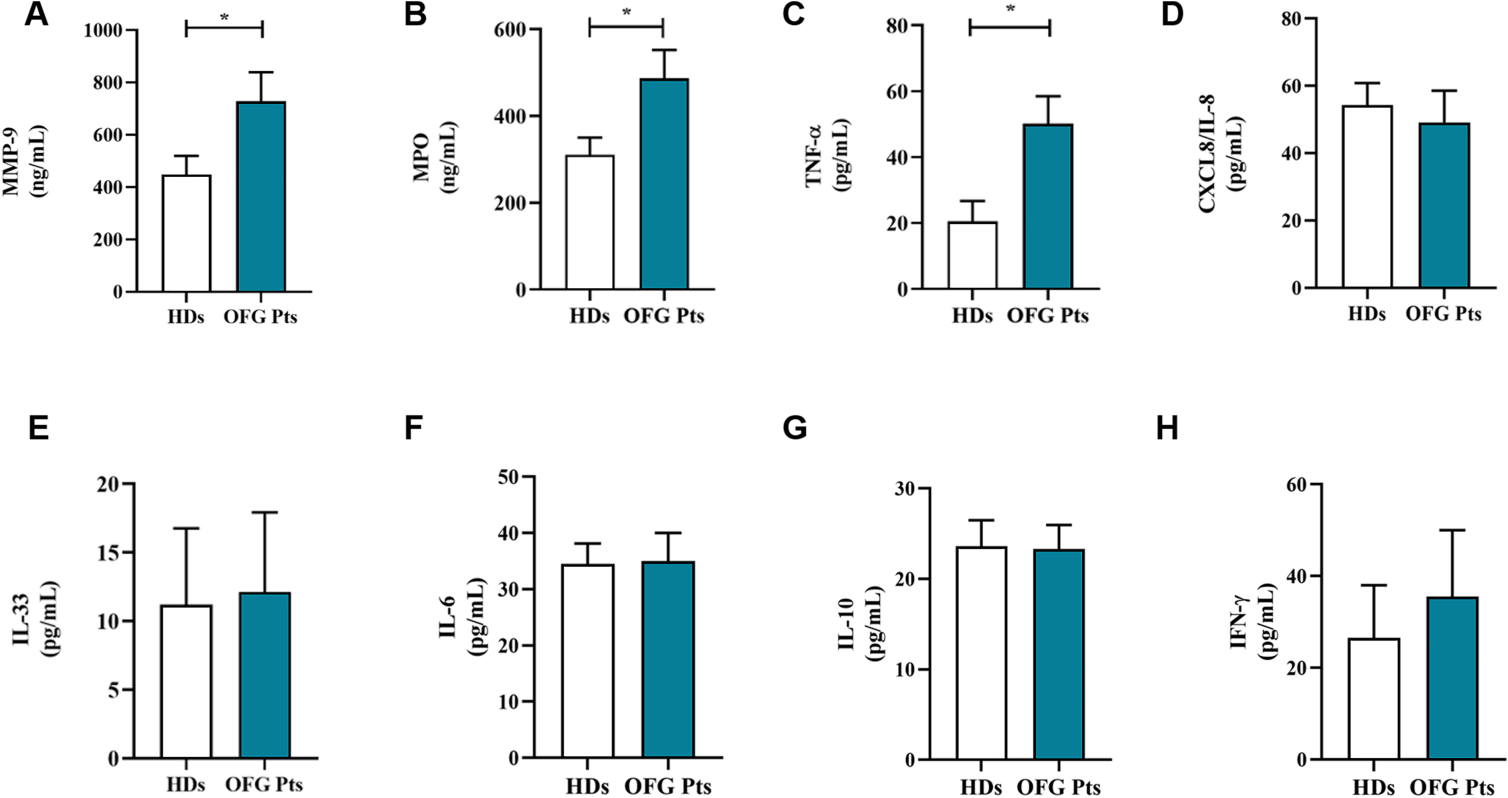
Serum concentrations of MMP-9 **(A)**, MPO **(B)**, TNF-α **(C)**, CXCL8/IL-8 **(D)**, IL-33 **(E)**, IL-6 **(F)**, IL-10 **(G)** and IFN-γ **(H)** in OFG patients (green bars) and HDs (white bars) were measured by ELISA. Results were expressed as mean ± SD. All the experiments were run in duplicate. *p < 0.05. Student’s t test or Mann-Whitney U test according to the parametric or nonparametric distribution of the variables.

**Figure 4:**
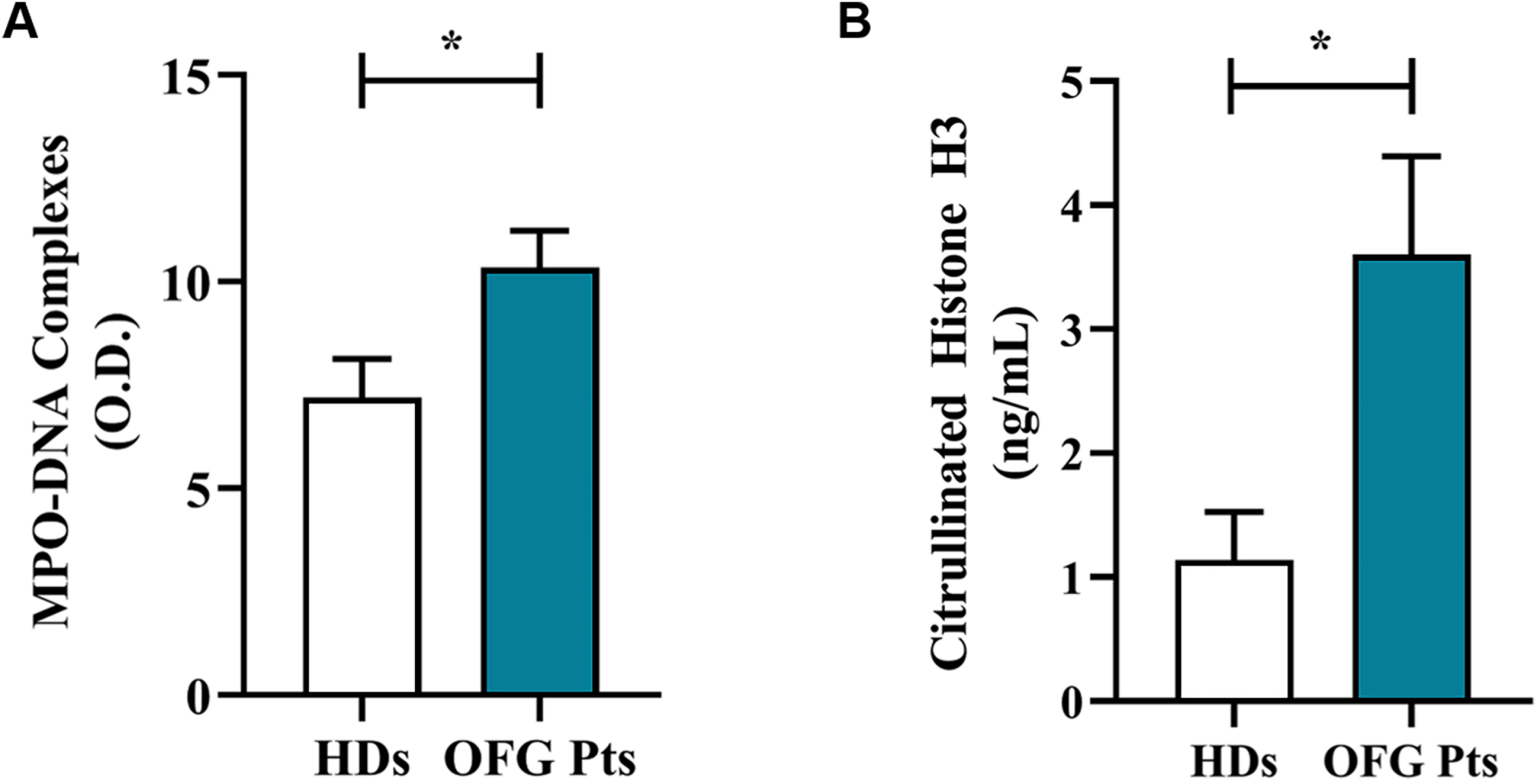
Serum levels of NET biomarkers, MPO-DNA Complex **(A)** and CitH3 **(B)** in OFG patients (green bars) and HDs (white bars) were measured by two different ELISA assays. Results were expressed as mean ± SD. All the experiments were run in duplicate. *p < 0.05. Student’s t test or Mann-Whitney U test according to the parametric or nonparametric distribution of the variables.

Serum levels of neutrophil-related mediators MMP-9 (**Figure 3A**), MPO (**Figure 3B**), TNF-α (**Figure 3C**), were increased in OFG patients compared to HDs. No differences were found in the circulating levels of CXCL8/IL-8 (**Figure 3D**), IL-33 (**Figure 3E**), IL-6 (**Figure 3F**), IL-10 (**Figure 3G**) and IFN-γ (**Figure 3H**) between OFG patients and HDs. Serum concentrations of MPO-DNA complexes (**Figure 4A**) and CitH3 (**Figure 4B**), two specific biomarkers for NET identification, were increased in OFG patients compared to HDs. Taken together, these data indicate that PMNs of OFG patients are activated *in vivo* to release neutrophil-derived mediators.

### PMNs activation in OFG patients

**Figure 5:**
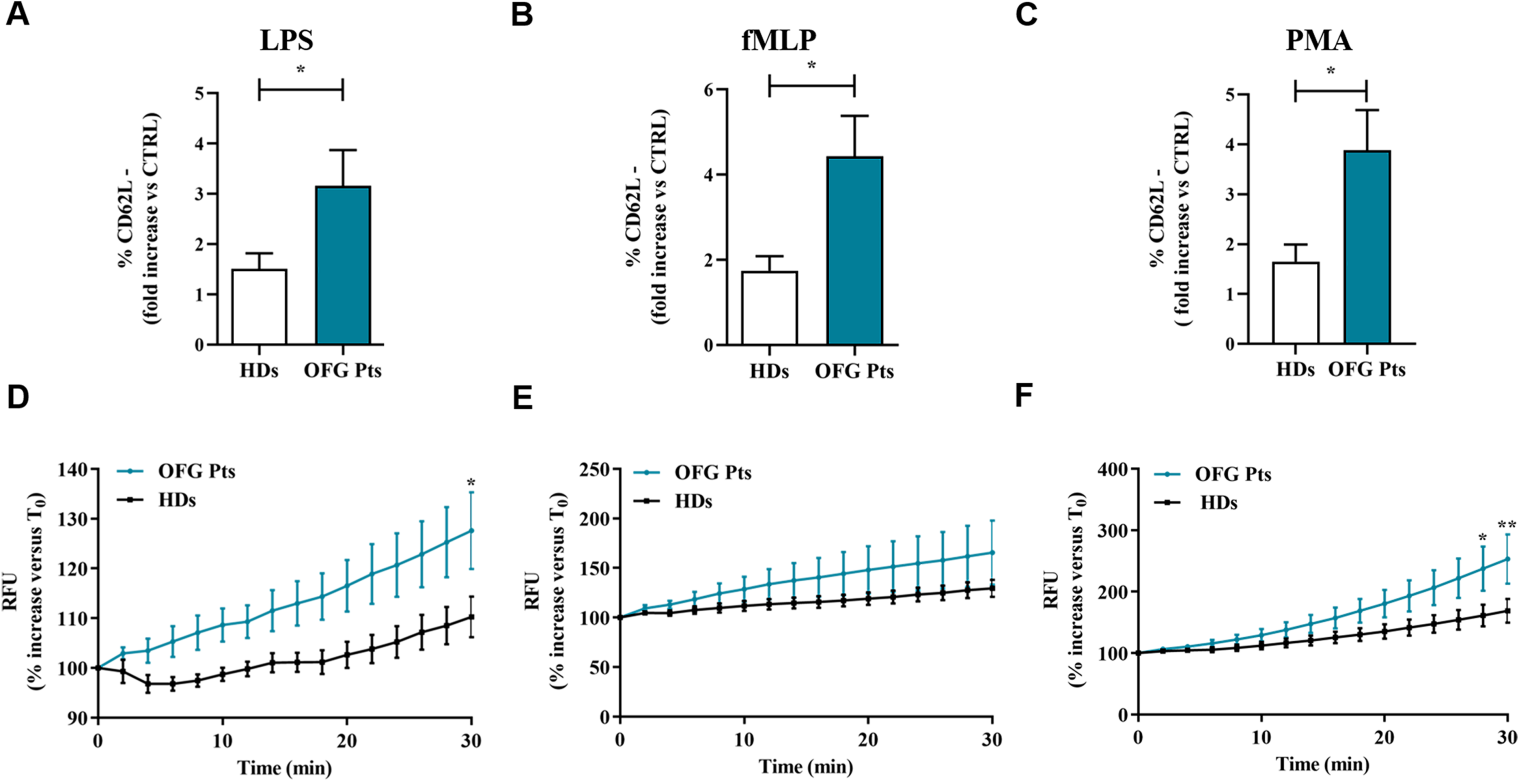
**(A-C)** PMNs (1.5 x 10^5^ cells) from peripheral blood of OFG patients (green bars) and HDs (white bars) were stimulated with complete medium, LPS (100 ng/mL) **(A)**, fMLP (1 µM) **(B)** or PMA (10 ng/mL) **(C)** for 1h at +37°C with 5% CO_2_, then stained for the neutrophil activation marker CD62L **(A-C)** and subjected to cytofluorimetric analysis. Results were expressed as mean ± SD. *p < 0.05. **(D-F)** PMNs (2 × 10^6^ cells) from peripheral blood of OFG patients (green line) and HDs (black line) were incubated (30 min, +37°C) with 2’,7’-dichlorodihydrofluorescein diacetate (H_2_DCFDA, 10 µM), washed with PBS and then stimulated with complete medium, LPS (100 ng/mL) **(D)**, fMLP (1 µM) **(E)** or PMA (10 ng/mL) **(F)**. Immediately after stimulation, PMNs were seeded in 96-well microplate (2 × 10^5^ cells/well) and were analyzed with a multimode microplate reader (EnSpire Multimode Plate reader, PerkinElmer), DCF fluorescence was measured for 30 min at 2 min intervals. The results were expressed as Relative Fluorescence Unit (RFU) and percentage increase *versus* time 0 (mean ± SD); All the experiments were run in duplicate. *p < 0.05; **p < 0.01.

To determine the activation status of PMNs isolated from of OFG patients, we evaluated the expression of one of the most widely used marker CD62L (L-selectin) on PMNs by flow cytometry (30). Based on the current understanding, under resting conditions, PMNs express CD62L, which is rapidly downregulated after cell activation (31). More in details, it has widely demonstrated that *in vitro* activation with various stimuli gives raise to CD62L shedding (32). PMNs from OFG patients showed higher percentages of activated PMNs (negative for CD62L: CD62L**^-^** cells), after stimulation with LPS (**Figure 5A**), fMLP (**Figure 5B**) and PMA (**Figure 5C**), compared to PMNs of HDs. Collectively these data indicate that the PMNs isolated from peripheral blood of OFG patients were more prone to be activated compared to their HDs counterpart.

### PMNs ROS production in OFG patients

To test whether PMNs activation leads to ROS production in OFG patients, we performed a H_2_DCF-DA ROS detection assay. **Figure 3** illustrates the kinetics (2 to 30 min) of the production of ROS induced by LPS (**Figure 5D**), fMLP (**Figure 5E**), and PMA (**Figure 5F**). LPS and PMA-induced ROS production in PMNs from OFG patients and HDs was evident after 25 minutes of stimulation. A similar trend was also observed when PMNs were stimulated with fMLP. No difference in ROS production was detected between OFG patients and HDs when RPMI was used as negative control (data not shown). These data indicate that PMNs from OFG patients, upon stimulation with LPS and PMA, showed an increased rate of ROS production compared to HDs.

## Discussion

In this study, we dissected the functional behavior of monocytes and PMNs in OFG patients. We found that these two immune cell types exhibited an activated phenotype in OFG patients compared to their counterpart of HDs. Monocytes and PMNs of OFG patients, when cultured with control medium, showed a cytokines release profile similar to that of HDs, with the exception of a higher TNF-α release from OFG-derived PMNs. Upon stimulation with different stimuli (i.e. LPS, fMLP and PMA), monocytes from OFG patients differentially released higher amounts of CXCL8/IL-8, IL-6, TNF-α and IL-33 compared to HDs. By contrast, when stimulated with some bacterial stimuli monocytes of OFG patients released lower quantities of IL-10, IFN-γ compared to HDs.

Similarly, upon stimulation with LPS, fMLP and PMA, PMNs of OFG patients differentially released large amount of TNF-α, MPO, IL-33 and ROS production compared to HDs PMNs. Conversely, CXCL8/IL-8 release was more pronounced in PMNs from HDs compared to PMNs of OFG patients. Finally, serum levels of MMP-9, MPO, TNF-α and NETs biomarkers (i.e. MPO-DNA complexes and CitH3) were higher in OFG patients compared to HDs.

Existing literature emphasizes the involvement of adaptative immunity in the pathogenesis of OFG (33) but the role of innate immune cells in OFG has not yet been widely explored. More in detail, clonal expansion of T cells and an enhance of related cytokines was demonstrated in OFG patients suggesting that could be responsible for triggering the granulomatous inflammation (34). The involvement of pro-inflammatory cytokines (e.g. IL-12 and TNF-α), derived from lymphocytes, was described in OFG patients, establishing that the microenvironment within the granulomatous tissue was predominantly Th1 (35). Our study provides novel insights into the functional characteristics of monocytes and neutrophils, key players in innate immune responses, in the context of OFG. Regarding monocytes, we have not found differences in spontaneous mediator release between monocytes from OFG patients and HDs, while the stimulation with LPS, fMLP, and PMA revealed differential responses. More in detail, monocytes from OFG patients exhibited increased release of pro-inflammatory mediators, including CXCL8/IL-8, IL-6, TNF-α, and IL-33, upon stimulation with the tested mediators compared to HDs monocytes. This heightened pro-inflammatory response aligns with the hypothesis that the implication of micro-organisms, especially bacteria, in the etiology of OFG (3). While inflammatory response induced in OFG monocytes is enhanced, the production of IL-10, an anti-inflammatory cytokine (36), induced by fMLP is reduced. IL-10 reduction by monocytes of OFG patients is in line with several studies, describing the impairment of this anti-inflammatory cytokine function in inflammatory conditions, such as OFG (37, 38). Despite, a Th1-dominant inflammatory environment characterized by elevated IFN-γ has been reported in OFG tissue biopsies (35), our study reveals a contrasting finding. More in detail, stimulated circulating monocytes from OFG patients released less IFN-γ compared to those from HDs. This discrepancy highlights the importance of considering both tissue-resident and systemic immune responses. It is conceivable that a compensatory mechanism is at play, whereby the reduced ability of circulating monocytes to release IFN-γ upon stimulation helps to balance the potentially elevated levels of this cytokine within the inflamed oral tissues. Alternatively, other cell types present in the OFG lesions, but not readily found in circulation, could be the primary source of IFN-γ within the tissue microenvironment. We then explored the role of PMNs in OFG patients. We found no differences in CXCL8/IL-8, IL-33 and MPO spontaneous release, between PMNs of OFG patients and HDs. TNF-α release was higher in PMNs of OFG patients compared to PMNs of HDs. Upon stimulation with LPS, fMLP and PMA, PMNs from OFG patients released more quantities of TNF-α and MPO compared to PMNs of HDs, and only when stimulated with fMLP, PMNs of OFG patients released higher levels of IL-33 compared to PMNs of HDs. A similar trend was seen in patients with Crohn’s disease, where PMNs of patients with this disease released higher levels of pro-inflammatory cytokines such as TNF-α, and when stimulated with LPS the release of these pro-inflammatory cytokines increased significantly compared to PMNs of HDs (39). By contrast, the CXCL8/IL-8 release by the PMNs of OFG patients was lower than HDs.

The involvement of some pro-inflammatory mediators (e.g. IL-6 and TNF-α), in salivary samples of patients affected by oral inflammatory diseases, has been well established (40, 41). Bronchoalveolar lavage fluid (BALF) in patients suffering from granulomatosis with polyangiitis (Wegener’s) (GPA) demonstrated an increased neutrophil cell count, elevated MPO, CXCL8/IL-8 and G-CSF concentrations and the presence of antineutrophil cytoplasmic antibodies, suggesting that the innate immune system, in particular PMNs, are crucial effector cells in this disease (42). Here we demonstrate that OFG patients display higher basal serum levels of some neutrophil-related mediators (MMP-9 and MPO) and of a pro-inflammatory cytokine such as TNF-α, confirming a role of innate immune system cells in this disease. Moreover, the serum NETs, assessed by MPO-DNA complexes and CitH3 (24, 25), are higher in OFG patients compared to HDs. The presence of NETs in inflammatory diseases (43, 44) and also in cancer-related inflammation (45) is well known, here for the first time we evaluated their presence and thus their involvement in OFG.

The activation status of a neutrophil can be evaluated both by the release of several mediators (e.g., cytokines/chemokines) and by CD62L shedding (21, 31, 32). Here we found that PMNs from OFG patients when stimulated with bacterial stimuli displayed higher percentages of activated PMNs (CD62L- cells) compared to PMNs purified from HDs. According to an increasing body of research, ROS have been linked to the pathophysiology of a high number of inflammatory diseases (46). The stimuli used in this study (LPS, fMLP and PMA) to stimulate neutrophils, are activators of ROS production in these cells through different pathways (47–49). Indeed, upon stimulation with LPS and PMA, PMNs of OFG patients displayed a higher ROS production compared to PMNs of HDs, in the presence of fMLP, OFG patients’ PMNs showed a slight increase in ROS production compared to HDs. Several investigators have proposed that patients affected by sarcoidosis and CD, in whom secondary OFG may occur, have elevated ROS production (50–52). Taken together these data indicate an activate phenotype of these cells in OFG patients.

**Figure 6:**
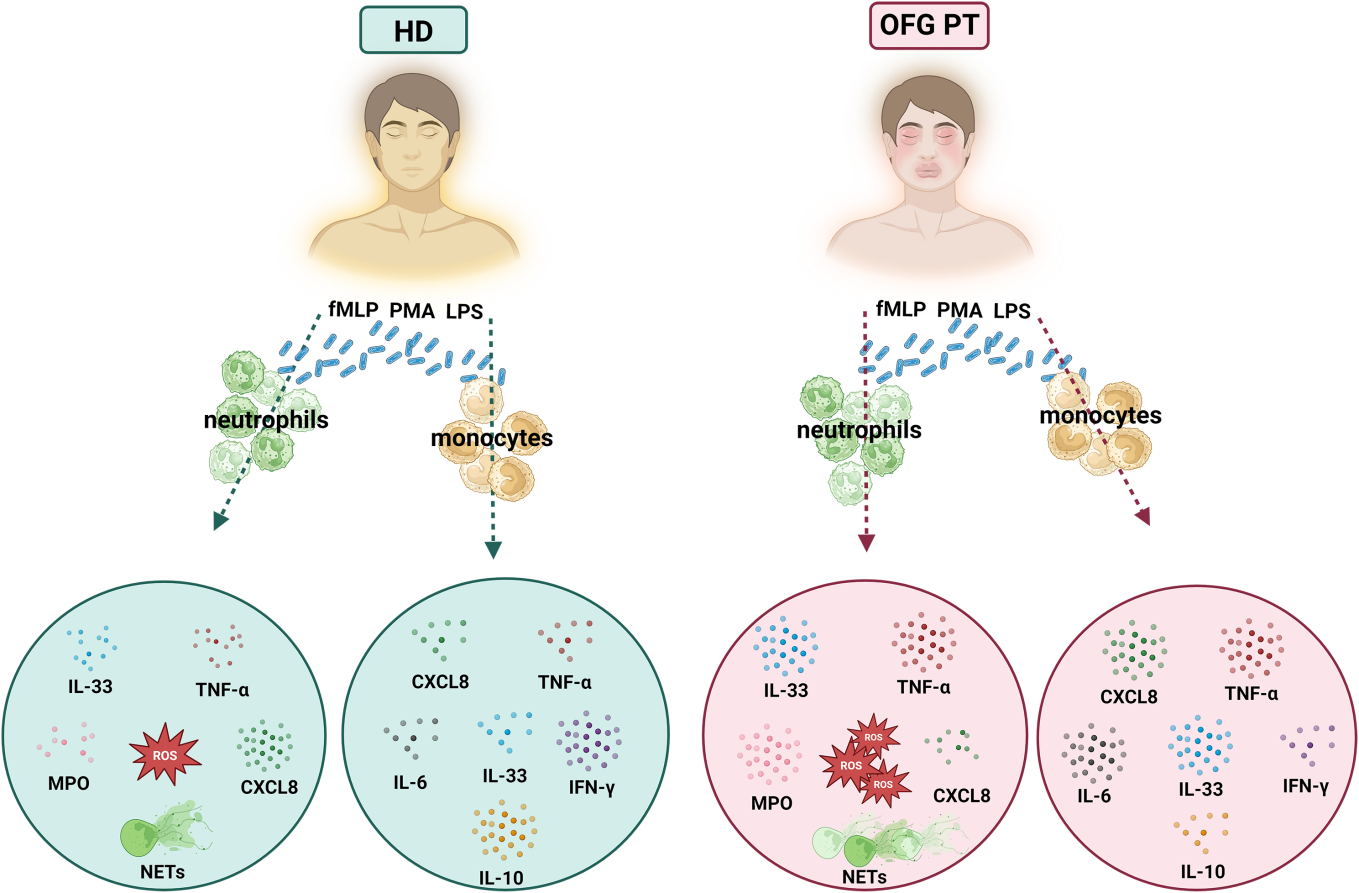
Graphical summary showing the differential activation of PMNs and monocytes of OFG patients and HDs under bacterial stimulation.

A graphical summary of the results is depicted in **Figure 6**.

In conclusion, these preliminary data, unfortunately on small sample size due to the rarity of disease, suggest that upon stimulation with various stimuli (bacterial and non-bacterial), monocytes and PMNs of OFG patients displayed an altered activation and phenotype compared to their counterpart of HDs that could predispose these patients to infections and inflammations. Unraveling the interplay between bacterial triggers and immune cell function in OFG will be necessary to elucidate mechanisms driving this complex disease and identify novel therapeutic targets for improved management of OFG patients.

## Data Availability

The raw data supporting the conclusions of this article will be made available by the authors, without undue reservation.
