# Supplementary material for: Differential activation of monocytes and PMNs in orofacial granulomatosis patients induced by bacterial and non-bacterial stimuli
Source: Front Immunol. 2025 May 6;16:1522495. doi: 10.3389/fimmu.2025.1522495 (PMC12088978; doi:10.3389/fimmu.2025.1522495)
Supplement: Supplementary file 1 [file Image1.pdf]

Supplementary Figure 1

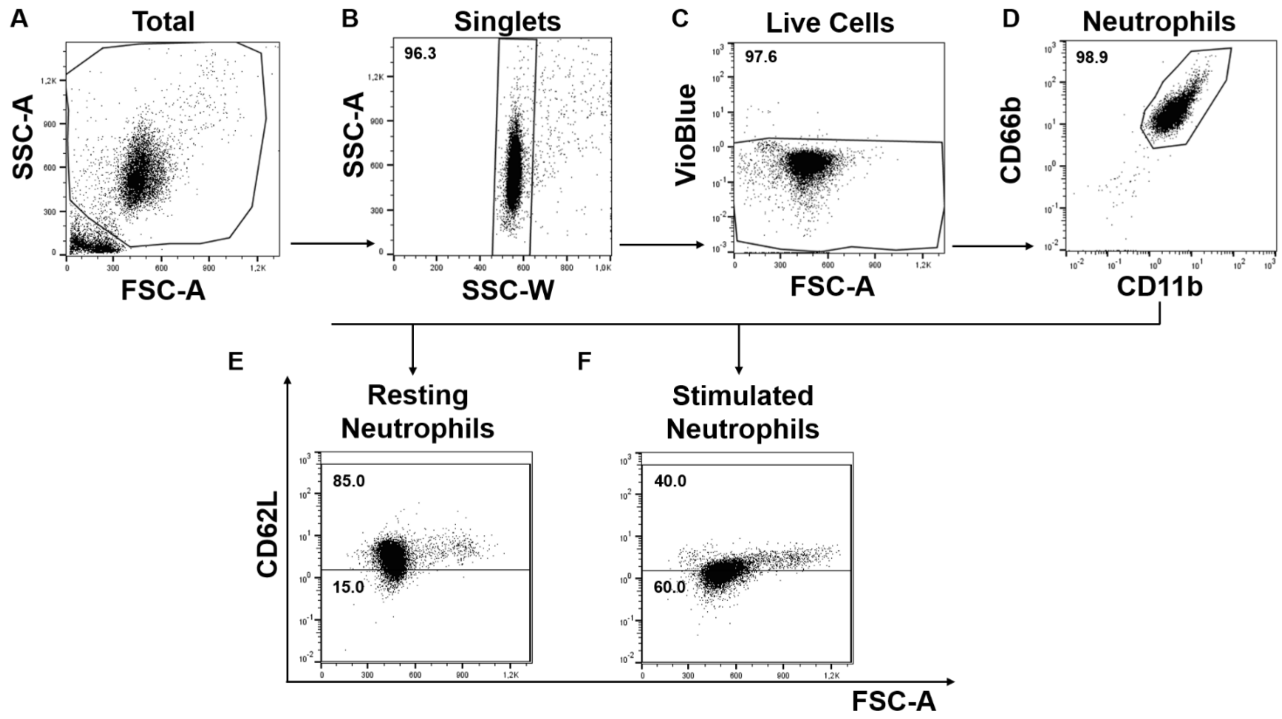

Supplementary Figure 1. Representative flow cytometric panels, with respect to the complete gating strategy for HDs and OFG patients, showing forward (FSC) and side scatter (SSC) of peripheral blood PMNs (**A, B**). Since Vioblue-positive cells included both dead cells and $CCR3^+$ cells (eosinophils), both cells were excluded based on a negative gate (**C**). PMNs were further identified as $CD66b^+$ $CD11b^+$ cells (**D**). Positive and negative PMNs for CD62L were gated on CD66b and CD11b double-positive cells (**E, F**).
